# Increasing Contraceptive Access for Hard-to-Reach Populations With Vouchers and Social Franchising in Uganda

**DOI:** 10.9745/GHSP-D-17-00065

**Published:** 2017-09-27

**Authors:** Benjamin Bellows, Anna Mackay, Antonia Dingle, Richard Tuyiragize, William Nnyombi, Aisha Dasgupta

**Affiliations:** a Population Council, Lusaka, Zambia.; b Marie Stopes International, London, UK.; c Independent consultant, London, UK.; d Marie Stopes Uganda, Kampala, Uganda.; e United Nations Population Division, New York, NY, USA.

## Abstract

From 2001 to 2011, modern contraceptive prevalence in Uganda increased from 18% to 26%. However, modern method use, in particular use of long-acting reversible contraceptives (LARCs) and permanent methods (PMs), remained low. In the 2011 Uganda Demographic and Health Survey, only 1 of 5 married women used a LARC or PM even though 34% indicated an unmet need for contraception. Between 2011 and 2014, a social franchise and family planning voucher program, supporting 400 private facilities to provide family planning counseling and broaden contraceptive choice by adding LARCs and PMs to the service mix, offered a voucher to enable poor women to access family planning services at franchised facilities. This study analyzes service trends and voucher client demographics and estimates the contribution of the program to increasing contraceptive prevalence in Uganda, using the Impact 2 model developed by Marie Stopes International. Between March 2011 and December 2014, 330,826 women received a family planning service using the voucher, of which 70% of voucher clients chose an implant and 25% chose an intrauterine device. The median age of voucher users was 28 years; 79% had no education or only a primary education; and 48% reported they were unemployed or a housewife. We estimated that by 2014, 280,000 of the approximately 8,600,000 women of reproductive age in Uganda were using a contraceptive method provided by the program and that 120,000 of the clients were “additional users” of contraception, contributing 1.4 percentage points to the national modern contraceptive prevalence rate. The combination of family planning vouchers and a franchise-based quality improvement initiative can leverage existing private health infrastructure to substantially expand family planning access and choice for disadvantaged populations and potentially improve contraceptive prevalence when scaled nationally.

## Abstract (precis)

Between 2011 and 2014, the program provided more than 330,000 family planning services, mostly to rural women in the informal sector with little or no education. 70% of the voucher clients chose an implant and 25% an intrauterine device.

## BACKGROUND

From 2001 to 2011, modern contraceptive prevalence in Uganda increased from 18% to 26% among married women of reproductive age.^1^ Despite these gains, the use of modern methods, in particular long-acting reversible contraceptives (LARCs) and permanent methods (PMs), remained quite low. In the 2011 Uganda Demographic and Health Survey (DHS), short-acting injections were the most common method used, and only 1 of 5 married contraceptive users reported using a LARC or PM.^1^ The 2011 Uganda DHS also reported that intrauterine devices (IUDs) were used by less than 1% of married women even though 34% of married women indicated an unmet need for family planning services.^1^ High modern contraceptive prevalence is associated with improved maternal health outcomes and a range of positive social outcomes.^2^^,^^3^

Access to a broad method mix is associated with higher contraceptive continuation rates, and higher levels of contraceptive use are associated with reductions in maternal and neonatal mortality and morbidity, a key development goal in low- and middle-income countries (LMICs).^4^^,^^5^ While the method mix has improved in many LMICs it remains skewed with short-acting modern contraceptives representing most of the observed increases in uptake in response to family planning program initiatives.^6^^,^^7^ Significant inequities and disparities remain in women's access to long-acting methods.^8^^,^^9^ LARCs remain effective over months and years, allowing women to delay, space, or limit births as they choose.^10^^,^^11^ For populations using LARCs, short-duration supply chain disruptions are less likely to increase risk of unintended pregnancies. Although initial costs for these methods are higher, the average cost over the period of use is often lower than less effective short-acting methods.^12^ LARC methods are often out of reach of the most vulnerable and marginalized women due to cost; fewer trained providers, in some settings; and lack of consumer awareness, among other barriers.^13^^–^^15^

For populations using LARCs, short-duration supply chain disruptions are less likely to increase risk of unintended pregnancies.

As in many LMICs, the barriers to family planning in Uganda exist at the patient, facility, health systems, and policy levels.^16^^,^^17^ Although policy and health system-level barriers can present significant challenges to LARC uptake, it is often high client out-of-pocket costs, lack of trained providers, and weak supply chains that present the most significant challenges in increasing access to and use of LARCs.^10^^,^^18^ Targeting provider subsidies and information to beneficiaries who would likely be unable to use the service in the absence of the intervention is essential to addressing these challenges. According to the 2011 Uganda DHS, over half of Ugandan contraceptive users access their method through the private sector. However, most private providers are not trained or equipped to provide a range of family planning services and, where LARCs are offered, financial barriers can prevent uptake.

High client out-of-pocket costs, lack of trained providers, and weak supply chains often present the most significant challenges in increasing access to and use of LARCs.

As described elsewhere, reproductive health voucher programs are designed around 3 key actors^19^:

1. A management agency and its community distribution agents
2. Beneficiaries voluntarily seeking family planning services
3. The family planning service provider

The management agency recruits and monitors community-based distributors who provide vouchers, which represent both a financial subsidy and community-based health education intervention, to disadvantaged or vulnerable clients. The management agency also establishes and maintains reimbursement to family planning facilities that provide contraceptive services at a predefined quality standard. In addition, programs can deploy independent agents to conduct verification and recurrent quality assurance checks. Voucher programs have been of increasing interest to policy makers and reproductive health care consumers in LMICs.^20^^–^^24^ A recent systematic review found that family planning voucher programs were associated with improved contraceptive uptake, reduced fertility, and increased family planning coverage among low-income populations. However, research gaps were noted, including a lack of study outcomes on family planning quality, unintended outcomes, client qualitative experiences, family planning voucher integration with health systems, and issues related to scale-up of the voucher approach.^25^

**FIGURE 1:**
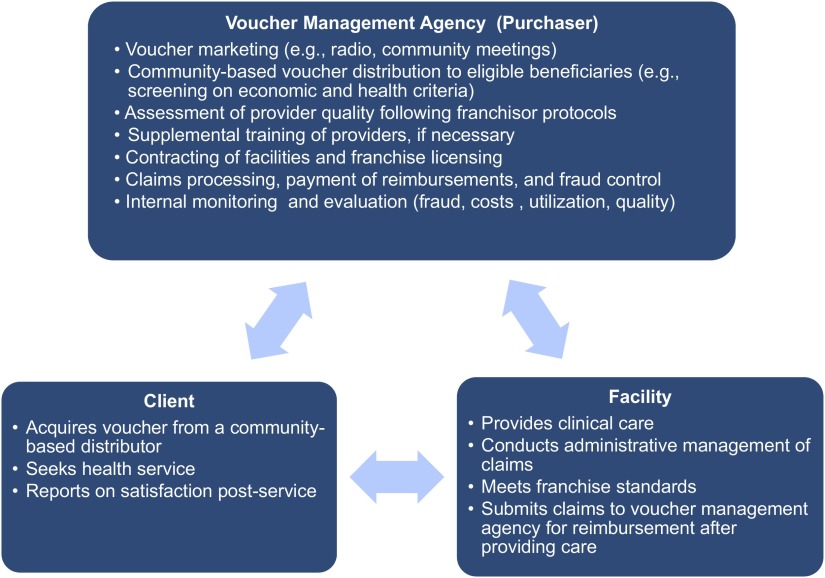
Marie Stopes Uganda Family Planning Social Franchise and Voucher Program Design and Functions

Between 2011 and 2014, Marie Stopes Uganda (MSU), a family planning NGO, used donor funds to increase access to LARCs and PMs through a combined social franchise and family planning voucher scheme (Figure 1). MSU trained and supported 400 private facilities across Uganda to provide high-quality family planning counseling and to increase family planning choice by adding LARCS and, in some cases, PMs to the available service mix through a partial franchise model. Through the model, MSU regulates and supports the franchisees' reproductive health and family planning services, allowing the franchisee to offer additional services.^10^ Before the initiative, few facilities provided LARC services due to perceived low consumer demand, limited supplies, and lack of provider skills. When they joined the MSU franchise network, facilities were introduced to standard quality assurance measures, provider training on LARC insertion and removal, regular supervision, intermittent quality checks, and service delivery area monitoring.^10^^,^^26^

Marie Stopes Uganda increased access to LARCs and PMs through a combined social franchise and family planning voucher scheme.

To reduce financial barriers to access, a voucher, with a consumer cost of approximately US$1, was introduced with a focus on poor women of reproductive age. A poverty grading tool—a short questionnaire that scores household assets and consumables to identify disadvantaged individuals—was used to determine eligibility for the voucher program and was administered during voucher distribution in Uganda.^27^^,^^28^ Adherence to the poverty grading tool criteria was verified during client follow-up surveys. MSU marketed the vouchers through different radio formats, community meetings and dramas, and direct-to-consumer activities. Clients accessed services using their vouchers during regular hours at social franchise outlets/partial franchisees. The voucher entitled consumers to family planning counseling, a method of their choice, and follow-up services, such as IUD removal, as needed. Although the voucher was intended to increase access to LARCs and PMs, as short-acting methods are usually available in the public sector, it was also redeemable for short-acting methods, to ensure family planning choice. Providers were reimbursed by MSU for voucher services provided after voucher claim vetting and validation.

The voucher entitled consumers to family planning counseling, a method of their choice, and follow-up services as needed.

## METHODS

This study presents service trends and voucher client demographics from the family planning voucher program in Uganda. Routine service delivery and client data were collected on every voucher client through a voucher management information system, with client demographic data recorded at the point of voucher distribution and cross-checked by the service provider. To reduce error and fraud, MSU conducted a medical plausibility review of all claims, data verification audits of sampled claims, and intermittent client follow-up checks. All data collection and analysis were conducted according to international principles of maintaining privacy and confidentiality of personal information.

Using the Impact 2 model developed by Marie Stopes International (MSI), the study estimated the health impact of the contraceptive services, such as pregnancies and maternal deaths averted, as well as contributions to contraceptive prevalence rate (CPR) growth and the contribution to national-level additional users of contraception in Uganda.

Impact 2 is a publicly available Excel-based model designed to use service provision data (http://mariestopes.org/impact-2).^29^ Impact 2 converts service data to the estimated number of contraceptive method users in a year, rather than the total number of clients who received services each year. Because LARCs and PMs offer multiple years of contraceptive coverage, the women who use these methods may not receive services annually. For example, some women who receive a LARC in 2012 could still be using the method in 2013, without receiving another service in 2013. The model factors in discontinuation of LARCs. For short-acting methods, the model estimates the number of services required for one year's worth of use. From the number of users of contraceptive methods, the model estimates the number of pregnancies averted and the resulting adverse health and economic outcomes averted, using best-available data on probabilities of these outcomes.

The model also takes into account data on who the program is reaching—for example, some women who are “new” to a provider may not be new to contraception—and estimates how these distinctions contribute to national-level additional users of contraception, in line with goals established by the global Family Planning 2020 (FP2020) initiative.^30^ While it is important that the social franchise and voucher program offers quality services and a fuller choice of methods, providing clients who were already using contraception from another provider with contraception services will not result in national-level increases in contraceptive use. Impact 2 addresses this by setting a “client profile,” which categorizes clients as:

- Adopters, who were not using a modern contraceptive method before receiving their service
- Continuing clients, who were already using a modern contraceptive method that they had received from the provider
- Provider-changers, who were already using modern contraception but previously received their method from a different provider

Impact 2 does not allow provider-changers to contribute to national-level growth in contraceptive use. Continuing clients are important to maintain the baseline of users, while adopters offset declines in user-numbers and contribute to national additional users. However, reliable data on the proportion of voucher clients who were adopters, continuers, and provider-changers were not available from the voucher client data set. Instead, the client profile used to generate Impact 2 additional user and CPR change data was estimated from client exit interviews carried out on a random sample of family planning clients using services from MSI's Social Franchise channel in Uganda in 2012 and 2013. The short, interviewer-administrated standardized questionnaire gathered information about the client's demographics and recent use of contraception. In the absence of a client exit interview survey for 2014, the 2013 client profile estimate was used. Because the family planning clients surveyed included both voucher and non-voucher users, and the exit interviews were not carried out in 2011 and 2014, the exit interview client profiles were proxies for the proportion of voucher clients who were adopters, continuers, and provider-changers. Exit interview data were used for the CPR change and additional user estimates only; all other findings were based on the routine voucher client data collected as part of the voucher management process.

After service data and the client profile were entered into the model, Impact 2 was run in “service life-span” mode to estimate the impact of services provided in a given year over the full life span of the methods—given that LARC and PM services will continue to provide contraceptive protection in future years. The service life-span concept applies to LARC and PM services only; for short-acting methods, there is no carry forward into future years. Using the service life-span mode ensures that the contribution of LARCs and PMs made in the first year is carried forward into subsequent years by including a modelled reduction in LARC use over time to reflect estimated discontinuation of methods use by current users.

## RESULTS

Between March 2011 and December 2014, 330,826 services were provided to women under the family planning voucher scheme in Uganda.

The median age for the family planning voucher users was 28 years (interquartile range [IQR], 23–32 years). Although gender was not recorded, gender-specific methods indicate that the vast majority of family planning voucher clients were women with a median of 3 living children. A majority (79.4%) of voucher clients had no education or only a primary education, and nearly half (47.6%) reported they were unemployed or self-described as a housewife. Almost a quarter (22.5%) of the clients were laborers or smallholder agriculturalists (Table 1).

**TABLE 1.** Family Planning Voucher Client Characteristics, 2011–2014 (N=330,826)

Characteristic	Value
Age,^a^ years, median (IQR)	28 (23–32)
No. of surviving children, median (IQR)	3 (2–5)
Education, No. (%)	
None	101,052 (30.6)
Some or completed primary	161,424 (48.8)
Some or completed secondary	61,449 (18.6)
Post-secondary	3,857 (1.2)
Missing	2,937 (0.8)
Occupation, No. (%)	
Unemployed/housewife	157,395 (47.6)
Agriculture/laborer	74,532 (22.5)
Professional	9,537 (2.9)
Other	13,702 (4.1)
Missing	75,660 (22.9)

Abbreviation: IQR, interquartile range.

^a^ Data on age were missing for 2,064 clients.

Most family planning voucher clients had no education or only a primary education, and nearly half reported they were unemployed or a housewife.

Although data on fertility preferences were limited, clients were asked about the number of currently living children they have and the number of children they desired to have in their lifetime. Approximately 6% reported having more children than they ideally wanted, 34% wanted no additional children, and 60% wanted to have 1 or more children in the future (data not shown).

Over half (58.6%) of clients heard about the voucher from either a community-based distributor (46.6%) or a health care worker (12.0%). A minority of clients heard about the voucher through a social contact, such as friends, family, or another satisfied user (15.4%); heard about the voucher through marketing and mobilization channels, such as behavior change communication, radio, banners, branding, and community mobilization (14.0%); or reported no information source (10.7%) (Table 2).

**TABLE 2.** Source of Information About the Voucher Program and Benefits (N=330,826)

Source	No. (%)
Community-based distributor	154,162 (46.6)
Health care worker	39,704 (12.0)
Missing/none	35,536 (10.7)
Friend/relative	30,750 (9.3)
Radio	29,156 (8.8)
Satisfied user	20,078 (6.1)
Behavior change communication promotions	12,365 (3.7)
Other	4,153 (1.3)
Branding	3,946 (1.2)
Community mobilization	976 (0.3)

**FIGURE 2:**
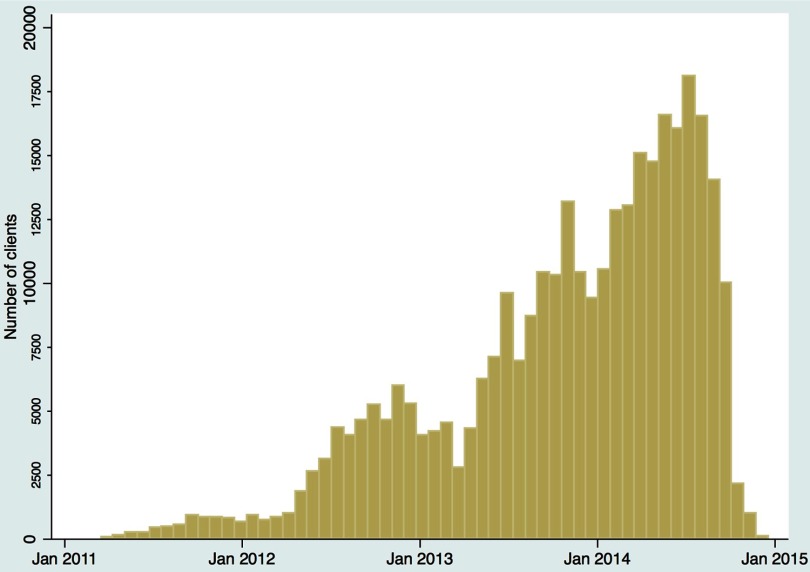
Date of Family Planning Voucher Clients' First Visit to a Marie Stopes Uganda Social Franchise Facility, January 2011 to January 2015

A majority (69.8%) of voucher clients used their voucher to receive an implant and 25.1% received an IUD (Table 3). Contraceptive uptake and client volume increased significantly over time. There appears to be a seasonal increase in October and November of each year, with a slight decrease in volume in late December and early January (Figure 2). This decrease may be attributed to facilities closing for the holidays and clients being preoccupied with other business.

**TABLE 3.** Family Planning Methods Selected by Voucher Clients, 2011–2014

	2011	2012	2013	2014	Total
	No. (%)	No. (%)	No. (%)	No. (%)	No. (%)
Implants	5,617 (89.4)	40,900 (85.8)	80,743 (70.8)	103,769 (63.7)	231,029 (69.8)
IUDs	660 (10.5)	5,741 (12.0)	27,720 (24.3)	48,822 (29.9)	82,943 (25.1)
Bi-tubal ligation	4 (0.1)	778 (1.6)	5,093 (4.5)	9,726 (5.9)	15,601 (4.7)
Vasectomy	1 (<0.1)	58 (0.1)	236 (0.2)	425 (0.3)	720 (0.2)
No method given	0 (0.0)	78 (0.2)	160 (0.1)	65 (<0.1)	303 (0.1)
Injectables	0 (0.0)	98 (0.2)	60 (0.1)	25 (<0.1)	183 (0.1)
Oral contraceptive pills	0 (0.0)	22 (0.1)	9 (<0.1)	6 (<0.1)	37 (<0.1)
Condoms	0 (0.0)	1 (<0.1)	3 (<0.1)	1 (<0.1)	5 (<0.1)
Emergency contraception	0 (0.0)	1 (<0.1)	3 (<0.1)	1 (<0.1)	5 (<0.1)
**Total**	**6,282 (100.0)**	**47,677 (100.0)**	**114,027 (100.0)**	**162,840 (100.0)**	**330,826 (100.0)**

Abbreviation: IUD, intrauterine device.

The estimated impacts of the program, as a result of the services provided in a given year, are presented in Table 4. Since LARCs and PMs provide multiple years of protection, some of these impacts will happen over multiple future years. For example, we estimated that the services provided in 2014 averted or will avert 218,000 unintended pregnancies and 520 maternal deaths, and save nearly US$14 million in direct health care costs.

**TABLE 4.** Estimated Impact of the Marie Stopes Uganda Social Franchise and Voucher Program, 2011–2014

	2011	2012	2013	2014
Unintended pregnancies averted	6,000	55,000	145,000	218,000
Maternal deaths averted	10	150	360	520
Direct health care costs saved (2014 USD)	429,000	3,400,000	9,100,000	13,800,000

Of the approximately 8,600,000 women of reproductive age in Uganda in 2014, we estimated that 280,000 were using a contraceptive method delivered through the franchise and voucher program. This estimate is derived from Impact 2, and includes those women who took up a contraceptive method in 2014, and the estimated portion of women who took up a LARC or PM in 2011–2013 and are estimated to be continuing to use that method in 2014. Applying the percentages of client profiles from the MSI exit interview results of 2012 and 2013, respectively (adopters 33%, 40%; continuers 32%, 22%; and provider-changers 36%, 37%), we estimate that 120,000 of the clients were “additional users” of contraception, which takes into account estimated adopters, discontinuation from previous years, and other dynamic factors in contraceptive use at the population level. We further estimate that this social franchise and voucher program added 1.4 percentage points to national mCPR between 2011 and 2014.

Of the 8.6 million women of reproductive age in Uganda in 2014, we estimated that 280,000 were using a contraceptive method delivered through the Marie Stopes Uganda franchise and voucher program.

**FIGURE 3:**
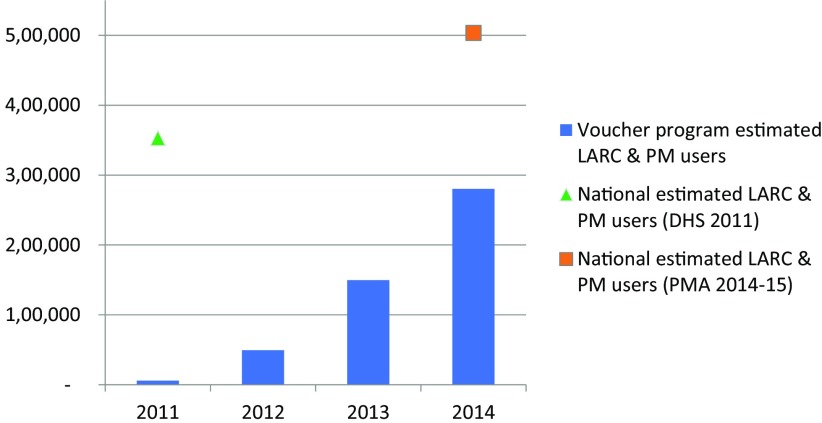
Marie Stopes Uganda Voucher Program Users Compared With National Estimates of LARC and PM Users, 2011–2014 Abbreviations: DHS, Demographic and Health Survey; LARC, long-acting reversible contraceptive; PM, permanent methods; PMA, Performance, Monitoring, and Accountability.

Figure 3 presents estimates of the number of family planning users from the voucher program by year, and puts this in the context of national LARC and PM user numbers derived from the DHS and the Performance, Management, and Accountability 2020 (PMA2020) surveys and population estimates.

## DISCUSSION

Over the nearly 4-year project period, the number of clients participating in the family planning social franchise and voucher program in Uganda increased substantially, with more than 330,000 services provided in total. The limited education level and high unemployment status of the voucher clients suggest that the voucher program was serving disadvantaged beneficiaries as intended. Nearly half of the clients heard about the family planning services from a community-based voucher distributor, which emphasizes the importance of mobilizing demand in the community to realize improvement in contraceptive access, particularly in rural areas. Successful rural mobilization via community-based agents was demonstrated in early voucher programs and, more recently, in generalized family planning initiatives.^31^^,^^32^

Over nearly 4 years, the social franchise and voucher program provided more than 330,000 family planning services.

The program's emphasis on reducing financial barriers to accessing LARCs was an important prerequisite to realizing World Health Organization tier-effectiveness counseling.^33^ If clients are aware that LARCs cost more than other methods, they are less likely to seek or request them. These findings are consistent with the literature that vouchers help to reduce the price barrier and likely empower clients to demand the most effective contraceptive method.^34^^,^^35^ Supply-side factors also contribute to improved LARC uptake, and the increase in uptake of implants in our analysis is consistent with other recent reports from social franchises in sub-Saharan Africa.^10^

Vouchers help reduce the price barrier to accessing LARCs and likely empower clients to demand the most effective contraceptive method.

By 2014, there were an estimated 280,000 women using contraception from the franchising and voucher program. To put this in context, there were an estimated 500,000 LARC and PM users nationally by 2014–2015.^36^ Based on preliminary findings from the 2016 Demographic and Health Survey, the modern method CPR among married women increased by 9 percentage points between 2011 and 2016, from 26% to 35%, and the LARC proportion of the method mix doubled during the same time period, from 12% to 22%.^37^ Furthermore, IUD use among married women increased threefold, from 0.5% to 1.5%.^37^ By reducing financial barriers to LARCs and PMs, the voucher and franchising program may have helped contribute to national-level increases in contraceptive prevalence: the estimated CPR increase from the voucher program of 1.4 percentage points represents 15% of Uganda's CPR increase between the 2011 and 2016 DHS surveys for modern methods among married women. Attribution from these program data is based on several assumptions. First, it is assumed that all other providers generally maintained their 2010 CPR contributions. Second, the extent to which any providers did not maintain their 2010 CPR contributions, the voucher program would have to offset that decline. Third, our analysis assumed a baseline of zero LARC users in 2010 at affiliated facilities; however, if the affiliated facilities were already providing LARCs and PMs prior to the start of the 2011 program, then at least part of the LARC and PM take-up seen in the voucher program might have taken place even in the absence of the program. The franchised facilities, however, were all newly trained on LARCs and PMs and thus the assumption that they did not provide LARCs and PMs before the voucher program is considered reasonable.

Operating the voucher scheme within a social franchise offers opportunities to undertake quality improvement interventions on the basis of voucher monitoring data. Voucher clients are engaged first at the community-based distribution point and again at the clinic visit. Because their contact details are available, they can be contacted later to gauge their satisfaction with and the quality of the services and methods they used. As can be seen from Table 3, while implants remained the most popular method throughout the project period, the proportion of women choosing IUDs increased significantly from 11% in 2012 to 30% in 2014. This likely can be attributed to a quality improvement intervention undertaken in 2013 that aimed to reduce supply-side barriers to method access and maximize client choice of method. Results from a 2013 voucher client follow-up survey indicated that many clients had not been fully counseled on IUDs. Follow-up interviews with providers revealed that franchisees lacked confidence on IUD counseling and provision. After retraining and mentoring of providers from mid-2013 onwards, increasing numbers of women chose IUDs, suggesting that franchisees were providing higher-quality counseling on IUDs as part of a comprehensive method mix, and women were better able to exercise method choice.

After retraining and mentoring providers, increasing numbers of women chose IUDs, suggesting that franchisees were providing higher-quality counseling on IUDs as part of a comprehensive method mix.

### Limitations

Because of the nature of the study, no comparison group was available. It is also possible that a secular increase in family planning utilization—in which a high number of disadvantaged individuals began to seek long-acting reversible contraception without relying on the voucher—could have occurred in Uganda during the program period. Additionally, the routine voucher data presented here may include recording errors by voucher distributors and providers. Finally, using social franchising client exit interview results as a proxy for voucher user client profiles may underestimate the proportion of voucher users who are family planning adopters, as non-voucher social franchise clients are more likely to return to using short-acting methods. However, the majority of social franchise clients between 2011 and 2014 were voucher users, so we consider the client exit interview results to be a reasonable proxy.

## CONCLUSION

The combination of family planning vouchers and a franchise-based quality improvement initiative can leverage existing private health infrastructure to substantially expand family planning access and choice for disadvantaged populations and potentially improve contraceptive prevalence when scaled nationally. Linking a voucher scheme with a social franchise also offers opportunities to undertake quality improvement interventions with providers and improve choice for women. Policy makers considering options to expand contraceptive coverage should consider testing a voucher initiative among private-sector franchises. For the public sector, the voucher system provides a mechanism by which to validate strategic purchasing of services for marginalized populations from franchised facilities.

## Notes

**Peer Reviewed**
